# Collaborative Referral Model to Achieve Hepatitis C Micro-Elimination in Methadone Maintenance Treatment Patients during the COVID-19 Pandemic

**DOI:** 10.3390/v14081637

**Published:** 2022-07-27

**Authors:** Chi-Ming Tai, Chun-Kai Huang, Te-Chang Changchien, Po-Chun Lin, Deng-Wu Wang, Ting-Ting Chang, Hsue-Wei Chan, Tzu-Haw Chen, Cheng-Hao Tseng, Chih-Cheng Chen, Chia-Ta Tsai, Yu-Ting Sie, Yung-Chieh Yen, Ming-Lung Yu

**Affiliations:** 1 Division of Gastroenterology and Hepatology, Department of Internal Medicine, E-Da Hospital, I-Shou University, Kaohsiung 84001, Taiwan; chimingtai@gmail.com (C.-M.T.); zeehou@yahoo.com.tw (T.-H.C.); 2 School of Medicine, College of Medicine, I-Shou University, Kaohsiung 84001, Taiwan; ed106531@edah.org.tw (T.-C.C.); ed108897@edah.org.tw (P.-C.L.); ed109635@edah.org.tw (D.-W.W.); ed104871@edah.org.tw (T.-T.C.); ed107263@edah.org.tw (H.-W.C.); chenhaug@gmail.com (C.-H.T.); city100c@gmail.com (C.-C.C.); ed107916@edah.org.tw (Y.-T.S.); 3 Division of Infectious Diseases, Department of Internal Medicine, E-Da Hospital, I-Shou University, Kaohsiung 84001, Taiwan; ed103536@edah.org.tw (C.-K.H.); protass0203@gmail.com (C.-T.T.); 4 Department of Infection Control, E-Da Hospital, I-Shou University, Kaohsiung 84001, Taiwan; 5 Department of Psychiatry, E-Da Hospital, I-Shou University, Kaohsiung 84001, Taiwan; 6 Division of Gastroenterology and Hepatology, Department of Internal Medicine, E-Da Cancer Hospital, I-Shou University, Kaohsiung 84001, Taiwan; 7 Hepatobiliary Division, Department of Internal Medicine, Kaohsiung Medical University Hospital, Kaohsiung 80756, Taiwan; fish6069@gmail.com; 8 School of Medicine and Hepatitis Research Center, College of Medicine, and Center for Liquid Biopsy and Cohort Research, Kaohsiung Medical University, Kaohsiung 80708, Taiwan; 9 School of Medicine, College of Medicine, National Sun Yat-sen University, Kaohsiung 80420, Taiwan

**Keywords:** hepatitis C, hepacivirus, methadone maintenance treatment, people who inject drugs, direct-acting antiviral agent, COVID-19

## Abstract

Although hepatitis C virus (HCV) prevails in patients receiving methadone maintenance treatment (MMT), most do not receive anti-HCV therapy. This single-center observational study aimed to achieve HCV micro-elimination at an MMT center during the COVID-19 pandemic using a collaborative referral model, which comprised a referral-for-diagnosis stage (January 2020 to August 2020) and an on-site-diagnosis stage (September 2020 to January 2021). A multidisciplinary team was established and all MMT center patients were enrolled. HCV micro-elimination was defined as >90% of HCV-infected patients diagnosed and >80% of HCV-viremic patients treated. A total of 305 MMT patients, including 275 (90.2%) anti-HCV seropositive patients, were enrolled. Among 189 HCV-infected patients needing referral, the accumulative percentage receiving HCV RNA testing increased from 93 (49.2%) at referral-for-diagnosis stage to 168 (88.9%) at on-site-diagnosis stage. Among 138 HCV-viremic patients, the accumulative percentage receiving direct-acting antiviral (DAA) therapy increased from 77 (55.8%) at referral-for-diagnosis stage to 129 (93.5%) at on-site-diagnosis stage. We achieved an HCV RNA testing rate of 92.4% (254/275), an HCV treatment rate of 95.8% (203/212) and a sustained virological response rate of 94.1% (191/203). The collaborative referral model is highly effective in HCV RNA testing and HCV treatment uptake among MMT patients, achieving HCV micro-elimination.

## 1. Introduction

People who inject drugs (PWID) are at high risk for HCV infection. Among the 71.1 million people living with hepatitis C virus (HCV) infection globally, 6.1 million people are PWID, representing 8.5% of all infections [1,2]. The prevalence of HCV infection is estimated to be 52.3% in PWID [3]. Higher prevalence of HCV infection has been reported in East and Southeast Asia, and Taiwan has a reported prevalence of higher than 90% [4]. The World Health Organization (WHO) has a goal of eliminating HCV as a public health threat by 2030 [5], and Taiwan aims to achieve this WHO goal by 2025 [6], particularly among PWID, which is a key population deserving special attention [7,8,9]. Globally, if the elevated HCV transmission risk was eliminated among PWID, an estimated 43% of incident HCV infections could be prevented [10].

Methadone maintenance treatment (MMT) is the most widely available treatment for addiction to heroin and other opiates. Because MMT patients must receive regular treatment to control the addiction, the MMT center becomes a useful entry point to HCV management for MMT patients [11]. Low levels of HCV testing and treatment uptake among PWID have been reported in the interferon (IFN) era. In addition to the lower effectiveness and significant patient side effects of IFN-based therapies, numerous barriers also exist at the patient, provider and healthcare system levels [12]. When considering the issue of HCV treatment, two types of MMT centers exist in Taiwan: pure MMT services and a general hospital that includes an MMT clinic and liver clinic within the same hospital. E-Da hospital is a tertiary referral hospital with both an MMT center and liver clinic at the same hospital site. We have previously reported the effectiveness of our integrated care for HCV treatment among MMT patients at a single center in the IFN era [13]. Anti-HCV-positive patients needed to be referred from this center to liver clinics for HCV RNA testing and anti-HCV treatment. Although linkage to care increased, treatment uptake with pegylated interferon/ribavirin (PEG-IFN/RBV) was disappointing. In addition to PEG-IFN/RBV-related barriers, other barriers in the care cascade, including patients’ lack of awareness or unwillingness to receive HCV treatment, and refusal to be referred if the MMT clinic and liver clinic visits were on different days, and patients with incomplete treatment due to being incarcerated or lost to follow-up, were also identified [13].

All-oral direct-acting antivirals (DAAs) are shown to have minimal side effects and high tolerability. Treatment with DAAs has led to a sustained virological response (SVR) rate in more than 95% of HCV populations, including PWID [14,15,16,17]. With the introduction of DAA regimens in HCV therapy, chronic HCV infection is able to be cured. Although estimated viremic HCV infections had decreased to 56.8 million people by the beginning of 2020, the number of patients initiated on treatment was estimated to have decreased in 2020 relative to 2019 [18]. In addition, Coronavirus Disease 2019 (COVID-19), which had was responsible for a pandemic in 2020, had also delayed many HCV elimination programs [19]. With DAA use, treatment-related barriers in PWID can be resolved; however, barriers in other steps of the care cascade persist. In this single-center observational study, we aimed to achieve HCV micro-elimination at an MMT center during the COVID-19 pandemic using a collaborative referral model.

## 2. Materials and Methods

### 2.1. MMT Patients

In this single-center observational study, all patients treated at the MMT center at E-Da hospital, Kaohsiung, Taiwan, between January 2020 and January 2021 were included. All PWID attending the MMT center for the first time were required to complete testing for HCV antibody (anti-HCV), hepatitis B virus surface antigen, human immunodeficiency virus (HIV) antibody and urine morphine levels, along with questionnaires regarding demographic data such as education, marital status and employment. MMT patients are required to visit MMT specialists every 4 weeks for adjustment of methadone dosage. Urine morphine and serum HIV antibody levels are checked every 12 and 24 weeks, respectively. Active drug users are defined as those who test positive for urine morphine during the 24 weeks before enrollment. The study was approved by the Ethical Committee of E-Da Hospital.

### 2.2. Collaboration of a Multidisciplinary Team

On 1 January 2019, the Taiwan National Health Insurance (NHI) authorized coverage of DAA prescriptions for all HCV-viremic Taiwanese citizens, but DAA therapy must be prescribed by hepatologists or infectious disease (ID) specialists. The HCV care cascade can be divided into several steps, including screening (anti-HCV testing), diagnosis (HCV RNA testing), linkage to care and anti-HCV treatment [20]. Although MMT, liver and ID clinics were located in the same hospital in the present study, referral of HCV-infected MMT patients to liver or ID clinics is still needed for HCV treatment. A multidisciplinary team was therefore established, including MMT specialists, hepatologists, ID specialists and case managers. The MMT case managers and specialists educated the patients and attempted to refer all anti-HCV-positive patients to liver or ID clinics, except for those who had records of undetectable HCV RNA within one year or evidence of SVR after being treated with PEG-IFN/RBV or DAA. According to the Taiwan NHI program, patients who receive DDA therapy should complete DAA therapy and testing for SVR. Because it took around 6 months from HCV RNA testing to DAA therapy and testing for SVR, patients who dropped out from our MMT center within 6 months due to being incarcerated or for personal reasons were excluded from referral. As shown in Table 1, several strategies were adopted to overcome barriers in the care cascade. A four-week strategy to reduce hospital visits and a simplified process to reduce outpatient waiting time were adopted to enhance patients’ acceptance for referral. In addition, a routine referral model was built that works sustainably. Because the patients at the MMT center are dynamic, this model ensures that the assessment of referral can be applied to all new patients at the first visit to our MMT center.

**Figure 1:**
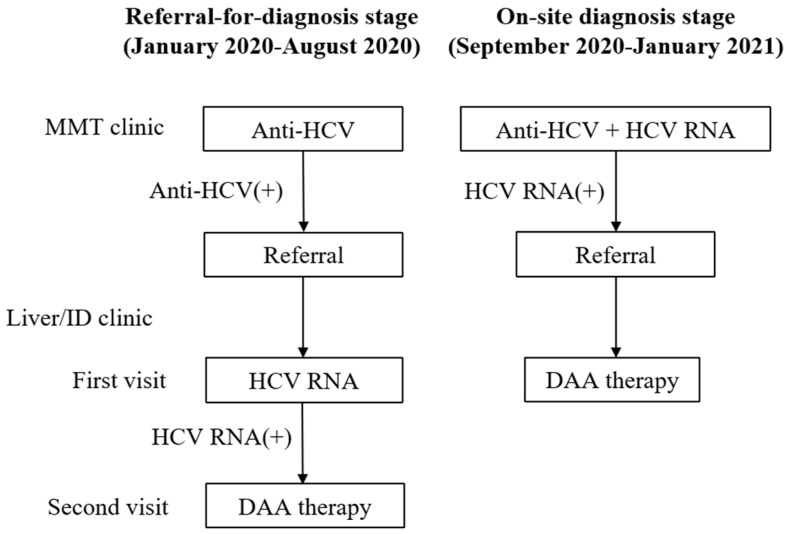
Two stages of the referral model. On-site diagnosis was applied to patients after referral-for-diagnosis failure.

The newly developed referral model was composed of two stages: a referral-for-diagnosis stage and an on-site-diagnosis stage (Figure 1). The referral-for-diagnosis stage started in January 2020 for all HCV-infected patients and ended in August 2020 because no more successful referrals were made. On-site diagnosis for all patients after referral-for-diagnosis failure started in September 2020 and ended in January 2021.

#### 2.2.1. Referral-for-Diagnosis Stage

At the referral-for-diagnosis stage, HCV RNA testing with reference to the HCV genotype was performed at patients’ first visits to the liver or ID clinic after successful referral. DAA therapy started at the next visit for those who had detectable HCV RNA. Patients were regarded as referral failures if they refused referral at least twice after education and recommendation of referral.

#### 2.2.2. On-Site-Diagnosis Stage

HCV RNA testing with reference to the HCV genotype for HCV-infected patients was performed at the MMT clinic at the on-site-diagnosis stage. HCV-viremic patients who were willing to receive DAA therapy were referred to the liver or ID clinic, and DAA therapy started at the first visit after successful referral.

### 2.3. Assessment of Treatment Responses

The HCV-viremic rate was defined as the proportion of HCV-viremic patients among the total HCV-infected patient population who received HCV RNA testing. The HCV treatment uptake rate was defined as the proportion of HCV-viremic patients who were being treated among the total number of HCV-viremic patients. Pan-genotypic regimens including sofosbuvir/ledipasvir (SOF/LDV) and glecaprevir/pibrentasvir (GLE/PIB) were the DAAs of choice for HCV treatment for PWID at our hospital. Complete treatment was defined as completion of the entire treatment regimen, usually 8 weeks and 12 weeks for GLE/PIB and SOF/LDV, respectively.

Serum HCV RNA was routinely assessed at the end of treatment and 12 weeks after treatment cessation. SVR12 was defined as undetectable HCV RNA 12 weeks after treatment cessation. Patients whose HCV RNA levels were not measured at the SVR time point were coded as non-SVR. Virologic failure and non-virologic failure were defined as detectable HCV RNA or lack of available HCV RNA data at post-treatment week 12, respectively. HCV micro-elimination was defined as HCV RNA testing performed in >90% of the HCV-infected patients and >80% of the HCV-viremic MMT patients treated [5].

### 2.4. Statistical Analysis

Data of continuous variables are presented as mean ± standard deviation, and categorical variables are presented as count and percentages (n, %). Serum HCV RNA level was expressed as the logarithmic transformation of the original values. SVR12 was estimated by intention-to-treat (ITT) analysis (all patients receiving DAAs) and per-protocol (PP) analysis (patients receiving DAA with HCV RNA data available at post-treatment week 12, excluding non-virological failures). Patients who received DAAs were divided into referral-for-diagnosis stage and on-site-diagnosis stage based on the period in which they received DAAs. Characteristics of these two groups were compared using Student’s *t*-test, chi-square test, or Fisher’s exact test, as appropriate. *p* < 0.05 was established as a statistically significant difference. All statistical analyses of data handling and associations were performed using SAS statistical software (version 9.4; SAS Institute, Inc., Cary, NC, USA).

## 3. Results

### 3.1. Characteristics of Participants

A total of 346 patients received MMT at the center between January 2020 and January 2021, and 41 patients who dropped out of the MMT center before two formal referrals for HCV treatment were excluded. Finally, 305 patients were enrolled in this observational study. The mean age of all patients was 49.5 years, and 275 (90.2%) patients were male. All patients completed HCV screening, with an overall anti-HCV seroprevalence of 90.2% (n = 275). HCV/HIV coinfection and HCV/HBV coinfection was found in 53 (19.3%) patients and 54 (19.6%) patients, respectively.

**Figure 2:**
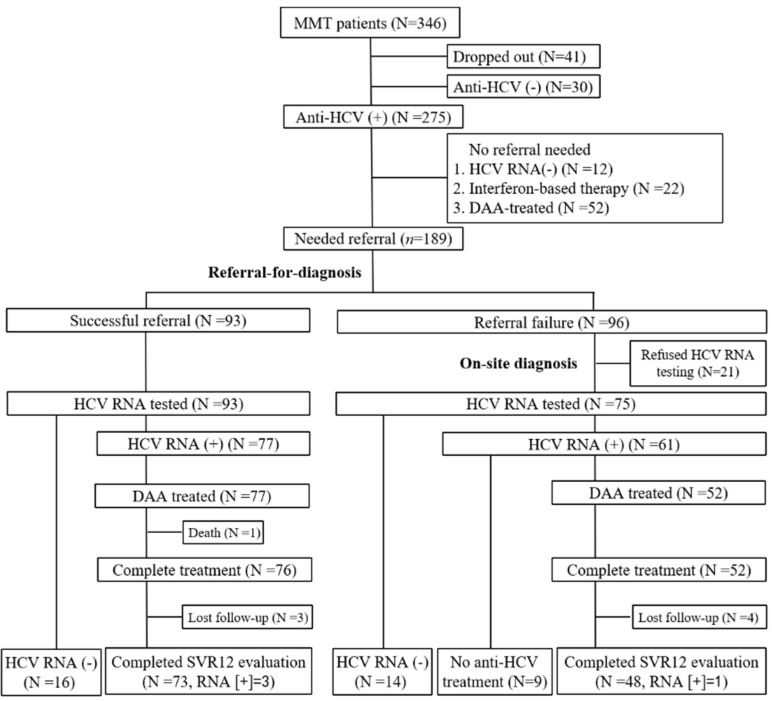
Flow chart of patients in the MMT center. MMT, methadone maintenance treatment; DAA, direct-acting antiviral; SVR, sustained virological response.

Of the 275 anti-HCV-seropositive patients, 86 did not need referral, including HCV RNA-seronegative (n = 12) or receiving successful antiviral therapy (n = 74; 22 with IFN-based therapy and 52 with DAA therapy) (Figure 2). Of the 189 HCV-infected patients who needed referral, successful referral was achieved in 93 and 66 patients in the referral-for-diagnosis stage and on-site-diagnosis stage, respectively. Finally, 30 patients were classified as referral failure, including 21 patients who refused HCV RNA testing and 9 HCV-viremic patients who were unwilling to receive DAA therapy (Figure 2). Reasons for refusal included unwillingness (n = 23), economic problems (n = 3), lack of awareness (n = 2), and health problems (n = 2). In summary, among the 275 HCV-infected patients at the MMT center, 31.3%, 33.8%, and 24.0% of patients completed the HCV care cascade before referral, at the referral-for-diagnosis stage, and at the on-site-diagnosis stage, respectively. Only 10.9% of the patients were classified as referral failure.

### 3.2. Effects of On-Site Diagnosis after Referral-for-Diagnosis Failure

Among 189 patients who needed referral, 93 (49.2%) patients accepted referral and received HCV RNA testing at the referral-for-diagnosis stage. Among these, 16 patients had undetectable HCV RNA and 77 (100%) HCV-viremic patients received DAA therapy (Figure 2). Compared with patients who refused referral at the referral-for-diagnosis stage, those who accepted referral had a higher proportion of HCV/HIV coinfection (8.3% vs. 22.6%, *p* = 0.007) (Table 2). Of the 96 patients who refused referral for HCV RNA testing at the referral-for-diagnosis stage, 75 patients (78.1%) agreed to receive HCV RNA testing at the on-site-diagnosis stage. Of the 61 HCV-viremic patients, 52 patients (85.2%) accepted referral for DAA therapy (Figure 2). Finally, of 189 HCV-infected patients, the cumulative percentage of patients receiving HCV RNA testing increased from 93 (49.2%) patients at the referral-for-diagnosis stage to 168 (88.9%) patients at the on-site-diagnosis stage. Of the 138 HCV-viremic patients, the cumulative percentage of patients receiving DAA therapy increased from 77 (55.8%) patients at the referral-for-diagnosis stage to 129 (93.5%) patients at the on-site-diagnosis stage.

### 3.3. Treatment Outcomes of 129 Patients Receiving DAA

Seventy-seven and 52 patients received DAA therapy at the referral-for-diagnosis stage and the on-site-diagnosis stage, respectively. Of the 129 patients who received DAA therapy, the mean HCV RNA level was 6.2 ± 0.9 log IU/mL, and the major genotypes were genotype 1 and genotype 6 in 47 (36.4%) patients and 53 (41.1%) patients, respectively. 

Treatment completion was achieved by 128 (99.2%) patients, except for one patient who died of pneumonia during treatment. Twelve patients did not have SVR 12, including four with virologic failure (non-response [n = 2] and relapse [n = 2]) and eight with non-virologic failure (stopped early [n = 1] and lost to follow-up [n = 7]). The HCV genotypes of the four patients with virologic failure were genotype 1, genotype 2, and genotype 6 in two, one, and one patients, respectively. No patients had cirrhosis or coinfection with HBV or HIV. Seven patients with non-virologic failure received complete treatment but lacked available HCV RNA data at post-treatment week 12 because of loss to follow-up. No patients had liver- or DAA-related serious adverse events that resulted in non-virologic failure. The rates of SVR12 were 90.7% (117/129) and 96.7% (117/121) in ITT and PP analysis, respectively (Table 3). Compared with patients who received DAA therapy at the on-site-diagnosis stage, those who received DAA therapy at the referral-for-diagnosis stage had a significantly higher proportion of HCV/HIV coinfection (24.7% vs. 3.8%, *p* = 0.002). No significant differences were found in HCVRNA levels, HCV genotypes, and SVR 12 between the two groups (Table 4).

### 3.4. Overall Performance of HCV Care Cascade in DAA Era 

**Figure 3:**
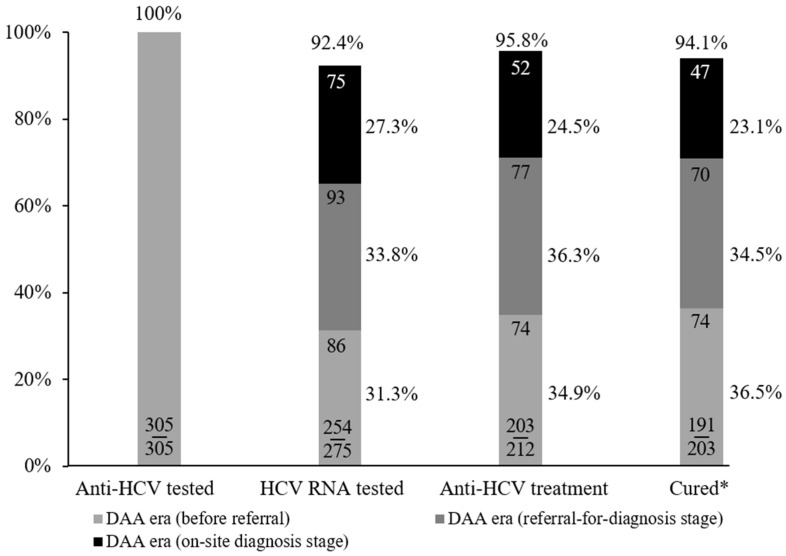
Overall performance in DAA era at the MMT center. * “Cured” was defined as undetectable HCV RNA 12 weeks and 24 weeks after the cessation of DAA treatment and PEG-IFN/RBV treatment, respectively. DAA, direct-acting antiviral; MMT, methadone maintenance treatment.

The overall performance at the MMT center is shown in Figure 3. Among 275 HCV-infected patients, 254 (92.4%) patients received HCV RNA testing. Of these, 212 (83.5%) patients were HCV-viremic, including 74, 77, and 61 patients at the pre-referral stage, at the referral-for-diagnosis stage, and at the on-site-diagnosis stage, respectively. The overall anti-HCV treatment uptake rate and SVR rate were 95.8% (203/212) and 94.1% (191/203), respectively.

## 4. Discussion

The results of the present study confirm that HCV micro-elimination was achieved at an MMT center in the DAA era, with an HCV RNA testing rate of 92.4%, an HCV treatment rate of 95.8%, and an SVR rate of 94.1%, even during the COVID-19 pandemic. In addition, on-site diagnosis was shown to increase MMT patients’ acceptance of HCV RNA testing and enhance treatment uptake after referral-for-diagnosis failure.

Starting the referral process with MMT case managers and MMT specialists, who have the closest contact and best relationship with MMT patients, is an essential step toward achieving a successful referral from the multidisciplinary team [21]. In addition to educating the patients, recording the reasons for refusal for referral may help to develop a plan to overcome the obstacles. The results of the present study showed that 65.1% of patients had completed HCV evaluation and treatment before referral and at the referral-for-diagnosis stage, and may therefore be regarded as an easy referral group; however, 10.1% of patients also refused referral. These data help to give an overview of the whole MMT center and to plan an overall referral strategy. Simplicity of referral is key to the scaling up of interventions and is widely considered to be a predictor of referral success among PWID [22]. MMT clinic and liver clinic visits on different days was one of the major reasons for referral failure in patients in the IFN era. A four-week strategy for patients to visit two clinics on the same day can solve this problem, and the number of liver or ID clinic visits also could be reduced to two and three times for patients receiving GLE/PIB and SOF/LDV, respectively. In addition, achieving HCV micro-elimination during the COVID-19 pandemic highlights the importance of our referral strategy. Patients are afraid of seeing a doctor during the COVID-19 pandemic, which has generally slowed HCV treatment. Although MMT patients must visit the MMT clinic every 4 weeks, they can still refuse to visit the liver or ID clinic. Therefore, scheduling appointments with the hepatologist or ID specialist on the same day as the MMT return visit may help to increase patients’ motivation and adherence to HCV treatment, and help to achieve HCV micro-elimination.

On-site diagnosis has been shown to enhance HCV testing among PWID and its effect among MMT patients has been obvious [23,24]. Adoption of on-site diagnosis, which improved the drawbacks of referral for diagnosis, was also one of the major reasons that HCV micro-elimination was achieved at our MMT center. The 78.1% of patients who refused referral at the referral-for-diagnosis stage agreed to receive HCV RNA testing at the on-site-diagnosis stage. In addition to simplifying the HCV care cascade, on-site diagnosis also significantly increased patients’ acceptance of referral and DAA therapy after referral-for-diagnosis failure. Because the only criterion for DAA therapy in Taiwan was detectable HCV RNA, patients’ motivation for DAA treatment will likely increase if they know that they can receive DAA therapy at the first visit after referral. Moreover, 16.5% of HCV-infected MMT patients had spontaneous HCV clearance, showing that on-site diagnosis also helped to select HCV-viremic patients who needed referral for DAA therapy.

On-site treatment, which may further simplify the HCV care cascade, has been shown to be more effective [25]. Although the Taiwan NHI authorized in October 2021 that DAAs can be prescribed by all doctors, including MMT specialists, we still adopted on-site diagnosis at our center, given the following considerations. First, the reasons of the remaining 30 patients who refused referral or treatment at our center were not related to stated barriers of referral for treatment. When we tried on-site treatment for these patients, treatment uptake did not increase. Second, the routine referral model was developed by integrating the daily practices of three departments, which did not increase the workload. MMT specialists must take on the additional training if they want to perform on-site treatment, which may become an implementation barrier. Third, the anti-HCV prevalence in PWID prisoners decreased from 91% in 2014 to 34.8% in 2019 by applying the strategy of safe injections in Taiwan [26], and case numbers of PWID also decreased in MMT centers. However, new HCV-infected MMT patients are still attending our center, and the routine referral model works well for HCV treatment in these patients. However, for institutions with a pure MMT service, HCV-infected MMT patients must be referred to other hospitals for DAA therapy, and referral is more difficult than that in our MMT center. On-site treatment will be most helpful in those institutions with a pure MMT service.

A significant finding at the MMT center was that 31.3% of MMT patients had already received HCV RNA testing or anti-HCV therapy before we started the referrals, which was higher than that in the IFN era (14.1%) [13]. MMT patients often have regular contact with MMT case managers and have increased opportunities to engage in HCV education, testing, and treatment [11]. In addition, we performed referrals for PEG-IFN/RBV treatment in 2015 and educated MMT patients about the importance of HCV treatment [13]. These efforts increased patients’ awareness, and MMT patients may actively seek HCV treatment when DAA therapy is available in Taiwan. PWID with HCV/HIV coinfection were reported to have a high prevalence of willingness to use DAAs, and treatment results were good [27,28]. In the present study, a high proportion of HCV/HIV coinfected patients also received referral or treatment, and 24 out of 53 HCV/HIV coinfected patients (45.3%) had already received HCV RNA testing or anti-HCV therapy before we started referrals. The major reason for this was that some HCV/HIV coinfected patients also received follow-up at ID clinics and received anti-HCV therapy. In addition, a higher proportion of patients with HCV/HIV coinfection also received referral and treatment uptake at the referral-for-diagnosis stage, and only three HCV/HIV coinfected patients (5.7%) refused referral or treatment at the end of the present study.

This study has several limitations. First, this was an observational study, and a randomized controlled trial was not conducted to compare the effectiveness between referral for diagnosis and on-site diagnosis. Other reasons unrelated to the model of care may have contributed to the increase in treatment uptake over time—for instance, increased awareness and desire among patients as they heard of or knew others who had been successfully treated and cured. Second, because treatment of reinfection after prior DAA treatment in Taiwan was not reimbursable by the Taiwan NHI at the time of the present study, patients with prior successful DAA treatment were not referred for HCV RNA testing. Third, it takes around 6 months to complete DAA therapy and follow-up in order to comply with the Taiwan NHI program. Patients who likely would drop out from the MMT center within 6 months were excluded from referral. These patients included those undergoing lawsuits and who may be incarcerated, those for whom the methadone treatment period may have ended, and/or patients who wished to transfer to another MMT center.

## 5. Conclusions

The collaborative referral model described herein is highly effective in HCV RNA testing and HCV treatment uptake among MMT patients. It helps to overcome the impact of the COVID-19 pandemic and is able to achieve HCV micro-elimination in an MMT center in the DAA era.

## Figures and Tables

**Table 1:** Strategies to overcome barriers in HCV care cascades of MMT patients.

**1. Integration of a multidisciplinary team to increase collaboration among three departments.**
A multidisciplinary team was established, including MMT specialists, hepatologists, ID specialists and case managers.
A consensus meeting was held before referrals began, to increase awareness of HCV treatment among MMT specialists and to avoid stigmatizing situations between the hepatologists or ID specialists and MMT patients. Regular meetings of the team were held monthly to overcome barriers to referral and treatment.
**2. Education of MMT patients to help overcome patients’ lack of awareness or unwillingness to accept HCV treatment.**
MMT case managers and MMT specialists educated the patients to improve their knowledge about HCV treatment, and also assessed the barriers to referral.
**3. Adoption of on-site diagnosis to replace r eferral-for-diagnosis method.**
On-site diagnosis increases MMT patients’ acceptance of HCV RNA testing and enhances treatment uptake after referral-for-diagnosis failure.
**4. Four-week strategy to reduce hospital visits.**
MMT patients were required to attend MMT clinic every 4 weeks. For patient convenience, hepatologist or ID specialist appointments were scheduled for HCV RNA testing or DAA therapy at the 4-week visits and usually on the same day as the MMT return visit. In addition, if patients forgot or neglected to return to the liver or ID clinic for DAA therapy or to check HCV RNA, MMT case managers reminded patients to complete the treatment course.
**5. Simplified process to reduce outpatient waiting time.**
Patients are often impatient while waiting to see a doctor or examination. Reducing outpatient waiting time helps to increase acceptance of referral and adherence to treatment.
**6. Collaboration with correctional institutions.**
If patients were incarcerated or dropped out from the MMT center before completing the DAA treatment course, we collaborated with other correctional institutions to complete the treatment course, usually including blood tests for SVR12.
**7. Routine referral model.**
Because patients at the MMT center are dynamic, the referral model ensures that assessment of referral can be applied to all new patients at first visit to the MMT center.

MMT, methadone maintenance treatment; DAA, direct-acting antiviral; ID, infectious disease; SVR, sustained virological response.

**Table 2:** Comparisons between patients who accepted referral and patients who refused referral at the referral-for-diagnosis stage.

Characteristics	Refused Referral	Accepted Referral	*p* Value
	(n = 96)	(n = 93)	
Age, years	49.1 ± 7.4	49.3 ± 7.5	0.879
Male sex	86 (89.6)	85 (91.4)	0.671
Education (senior high school or higher)	42 (43.8)	41 (44.1)	0.963
Employment	70 (72.9)	71 (76.3)	0.588
Alcohol consumption	23 (24.0)	28 (30.1)	0.341
Smoking	87 (90.6)	85 (91.4)	0.853
HBV coinfection	17 (17.7)	21 (22.6)	0.403
HIV coinfection	8 (8.3)	21 (22.6)	0.007
Liver cirrhosis	3 (3.1)	7 (7.5)	0.177
Prior interferon experience	1 (1.0)	5 (5.4)	0.089
MMT duration, year	4.7 ± 4.1	5.0 ± 3.9	0.350
Active drug user	74 (77.1)	69 (74.2)	0.643

Values expressed as mean ± standard deviation or sample size and proportion (%). HBV, hepatitis B virus; HIV, human immunodeficiency virus; MMT, methadone maintenance treatment.

**Table 3:** Treatment outcomes of 129 patients receiving DAA.

	n/N (%)
DAA regimens	
SOF/LDV	77/129 (59.7)
GLE/PIB	52/129 (40.3)
Complete treatment	128/129 (99.2)
EOTVR	124/129 (96.1)
SVR12 (ITT)	117/129 (90.7)
SVR12 (PP)	117/121 (96.7)
Reasons for non-SVR12	n = 12
Virologic failure	
Non-response	2
Relapse	2
Non-virologic failure	
Stopped early	1
Lost to follow-up	7

DAA, direct-acting antiviral; SOF, sofosbuvir; LDV, ledipasvir; GLE, glecaprevir; PIB, pibrentasvir; EOTVR, end-of-treatment viral response; SVR, sustained viral response; ITT, intention-to-treat; PP, per-protocol.

**Table 4:** Comparisons between patients receiving DAA treatment at the referral-for-diagnosis stage and on-site-diagnosis stage.

Characteristics	Referral-for-Diagnosis Stage	On-Site Diagnosis Stage	*p* Value
	(n = 77)	(n = 52)	
Age, years	48.8 ± 7.7	49.7 ± 7.2	0.607
Male sex	77 (90.9)	46 (88.5)	0.651
Education (senior high school or higher)	34 (44.2)	23 (44.2)	0.993
Employment	57 (74.0)	40 (76.9)	0.709
Alcohol consumption	25 (32.5)	11 (21.2)	0.160
Smoking	73 (94.8)	50 (96.2)	0.721
HBV coinfection	11 (14.3)	5 (9.6)	0.430
HIV coinfection	19 (24.7)	2 (3.8)	0.002
Liver cirrhosis	7 (9.1)	3 (5.8)	0.489
Prior interferon experience	5 (6.5)	1 (1.9)	0.227
MMT duration, year	4.6 ± 3.8	4.5 ± 4.3	0.190
Active drug user	54 (70.1)	40 (76.9)	0.395
AST, IU/L	58.6 ± 45.3	54.6 ± 50.1	0.706
ALT, IU/L	62.6 ± 57.9	57.2 ± 39.4	0.224
White cell count ×103/μL	6.3 ± 2.1	6.9 ± 1.9	0.400
Hemoglobin, g/dL	14.1 ± 1.9	14.6 ± 1.4	0.186
Platelet count, ×103/μL	195.5 ± 79.0	194.9 ± 72.1	0.754
Albumin, g/dL	4.2 ± 0.4	4.3 ± 0.4	0.878
Total bilirubin, mg/dL	0.6 ± 0.3	0.7 ± 0.3	0.158
Baseline HCVRNA, log IU	6.1 ± 1.0	6.3 ± 0.7	0.107
HCV genotype,			0.458
1/2/3/6	27 (35.1)/12 (15.6)/5 (6.5)/29 (37.7)	20 (38.5)/8 (15.4)/0 (0)/24 (46.2)	
1 + 2/unclassified	2 (2.6)/2 (2.6)	0 (0)	
SVR 12			
ITT	70/77 (90.9)	47/52 (90.4)	0.920
PP	70/73 (95.9)	47/48 (97.9)	0.542

Values expressed as mean ± standard deviation or sample size and proportion (%). DAA, direct-acting antiviral; HCV, hepatitis C virus; HBV, hepatitis B virus; HIV, human immunodeficiency virus; MMT, methadone maintenance treatment; AST, aspartate aminotransferase; ALT, alanine aminotransferase; SVR, sustained viral response; ITT, intention-to-treat; PP, per-protocol.

## Data Availability

All the data can be obtained upon request to the corresponding authors.
